# Clinical epidemiological characteristics of mycetoma in Eastern Sennar locality, Sennar State, Sudan

**DOI:** 10.1371/journal.pntd.0009847

**Published:** 2021-12-13

**Authors:** Rowa Hassan, Kebede Deribe, Ahmed Hassan Fahal, Melanie Newport, Sahar Bakhiet

**Affiliations:** 1 Mycetoma Research Centre, University of Khartoum, Khartoum, Sudan; 2 Department of Global Health and Infection, Brighton and Sussex Medical School, Brighton, United Kingdom; 3 Children’s Investment Fund Foundation, Addis Ababa, Ethiopia; 4 School of Public Health, College of Health Sciences, Addis Ababa University, Addis Ababa, Ethiopia; University Hospitals Sussex NHS Foundation Trust, UNITED KINGDOM

## Abstract

Mycetoma epidemiological features remain uncharacterised. Few studies have been conducted in a community-based setting to explore the epidemiological features and risk factors for mycetoma in Sudan. To bridge this gap, this study was conducted in Eastern Sennar Locality, Sennar State, Sudan, to report the clinical, epidemiological characteristics of mycetoma patients and the disease burden in the state.

We used cluster sampling; sixty villages were randomly selected across the locality’s five administrative units, and a household-to-household survey was conducted. We collected data using pre-designed questionnaires at the community, household, and individual levels. We performed descriptive analyses of the data and produced prevalence maps using ArcGIS 10.5 ([ESRI] Inc., Redlands CA, USA).

A total of 41,176 individuals were surveyed, and 359 mycetoma patients were identified. The overall prevalence of mycetoma was 0.87% (95%CI = 0.78–0.97%), the prevalence among males was 0.83% (95%CI = 0.71–0.96%), and females 0.92% (95% CI = 0.79–1.06%). Individuals in the age group 31–45 years had the highest prevalence among the different age groups (1.52%, 95% CI = 1.23–1.86%). The prevalence map showed patients clustered within the central and north-eastern part of the locality, while villages in the south-western part had few or no cases.

In conclusion, this clinical epidemiological study is pioneering and shows that mycetoma is prevalent in certain parts of Sudan. This data obtained will support the design of measures to reduce the disease burden in the state. The survey procedures and protocols can be adopted for further studies in Sudan and beyond.

## Author summary

Mycetoma is a neglected tropical disease documented in many countries around the world, yet few community-based studies have been performed investigating the disease burden, clinical epidemiological features, and risk factors. The overall prevalence of mycetoma in the study area was 0.87%, and the prevalence of mycetoma among males and females was almost the same. Individuals in the age group 31–54 years had the highest prevalence among all age groups. Married individuals had a higher prevalence when compared to unmarried individuals. Prevalence among those who could not read or write was 1.24% compared to 0.87% in those who could. History of trauma increased the prevalence to 1.63%, while individuals with no history of trauma had a prevalence of 0.70%. There were nine villages with no cases and an average of seven cases per village across the other 51 villages (range 1–39). The prevalence map showed patients clustered within the central and north-eastern part of the locality, while villages in the south-western part had few or no cases. The disease prevalence reported here may be more accurate as it was generated from a large study population. Further epidemiological studies are needed to determine mycetoma prevalence in Sudan and bridge the gaps in our understanding of the epidemiology of mycetoma.

## Introduction

Mycetoma is a neglected tropical disease (NTD) that is widely endemic in tropical and subtropical regions [1]. It usually presents mostly in the feet with painless soft tissue swelling associated with multiple sinus tracts formation and discharge of grains [2]. Mycetoma is caused by bacterial and fungal organisms, namely actinomycetoma and eumycetoma respectively, it spreads to the skin, deep structures, and bone, leading to deformities and disability [3,4]. It is speculated that the organisms are introduced to the body through a minor wound caused by sharp objects such as thorns, particularly acacia tree thorns [5]. Mycetoma affects poor people in remote rural communities, especially those working directly with the environment and animals such as farmers who tend to crop and livestock and shepherds[6]. Young male adults are a commonly affected group [7,8].

Diagnosis is usually made by the distinctive clinical presentation of mycetoma followed by different imaging techniques to confirm the disease presence and extent [9,10]. A biopsy is taken from the lesion site for histopathological examination or fine-needle aspirates for cytological examination to identify the causative organism. Recently, molecular identification techniques were introduced [11]. Management of mycetoma depends on the type of disease. Eumycetoma is treated with a combination of medical treatment and surgical excision, while actinomycetoma cases respond well to medical therapy alone[12].

The epidemiological features associated with mycetoma are not well described. The disease prevalence and incidence are still unknown worldwide. In Sudan, there are few studies reporting prevalence and incidence[13]. Although mycetoma was first reported in 1842, Abbott’s first attempt to determine its prevalence was in 1956. He studied 1321 mycetoma cases from different parts of Sudan, and he reported a disease prevalence of 0.51% among hospital patients seen in Khartoum during a study period of 36 months. He reported higher prevalence in Atbara, Ed Dueim, and Wad Madani cities (within central Sudan states) with estimates of 0.92%, 0.93% and 1.18%, respectively [14]. In 2014, Fahal and colleagues conducted a study in a village in White Nile State, Sudan, to determine the burden of mycetoma, and reported a prevalence of 1.45% [15].

Only a few community-based studies have investigated the disease burden, clinical epidemiological features and risk factors for mycetoma. This study uses data collected from a large survey in Eastern Sennar Locality, Sennar State, to investigate the disease epidemiological characteristics.

## Methods and materials

### Ethical statement

Ethics approval for this study was obtained from the Mycetoma Research Centre, Khartoum, Sudan IRB (Approval no. SUH 11/12/2018) and from the BSMS Research Governance and Ethics Committee (ER/BSMS435/1). Written informed consent was obtained from each adult patient and parents or guardians of the population under 18 years old. Confirmed mycetoma cases were referred for management at Wad Onsa Regional Mycetoma Centre or the Mycetoma Research Centre (MRC).

### Study setting

**Fig 1:**
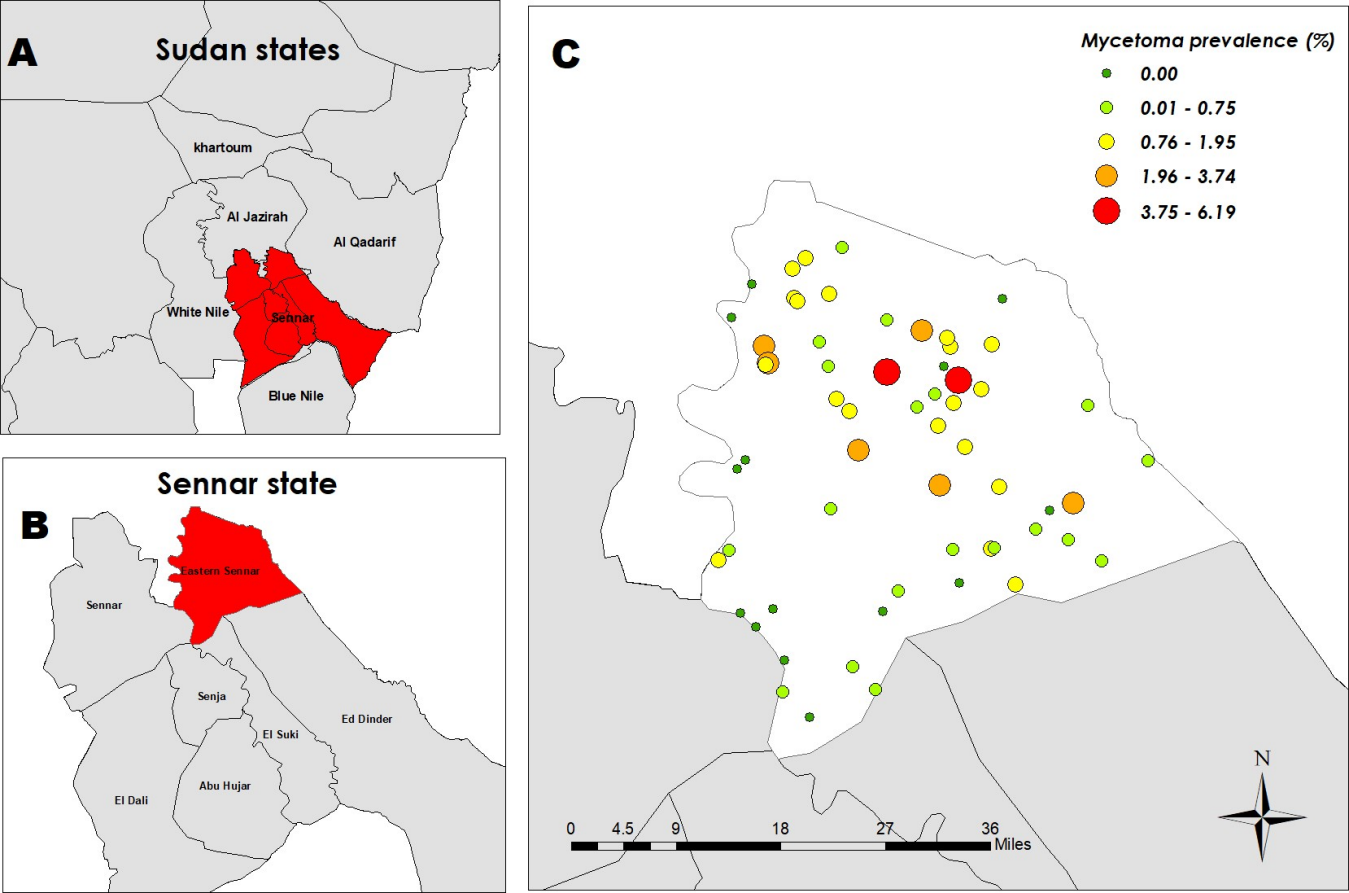
Geographical distribution of mycetoma in the sixty villages in Eastern Sennar Locality, Sennar State, with a total population of 41,176 individuals. Base map of Sudan link: https://www.diva-gis.org/datadown. A-Sudan states map with Sennar State in red. B-Sennar State Localities with Eastern Sennar Locality in red. C-Geographical distribution of mycetoma in Eastern Sennar locality.

Eastern Sennar Locality is one of seven localities in Sennar State. This state is situated in the southeast part of Sudan. Jazeera State in the north borders it, the Blue Nile State in the south, Al-Gadaref State in the east and the White Nile State & the Upper Nile State of South Sudan in the west [16]. Eastern Sennar Locality is subdivided into five administrative units: Wad Alabbas, Wad Onsa, Wad Taktok, Elreif Elshargi and Doba. (Fig 1A and 1B)

### Sampling strategy and participant selection

A cross-sectional community-based study was conducted. Cluster sampling was used to select sixty villages randomly within the five administrative units of Eastern Sennar Locality.

Total coverage of these villages with a household-to-household survey was conducted. Data were collected using pre-designed validated questionnaires at three levels: community, household, and individual. All individuals living in the selected villages who provided written informed consent were included in the study.

The survey team consisted of a coordinator responsible for organising the survey activities and communication with village leaders and a medical doctor responsible for obtaining the written consent, performing the clinical examination and interviewing individuals within the household. Also, the team had a village guide who was responsible for the facilitation of movement within the village and communications with villagers. The teams visited the villages before the actual day of the survey, where the local leaders and community representatives were informed of the survey objectives, then teams identified a suitable day and time for the survey process and recorded geo-coordinates of the village using a GPS device. All households were then visited. Informed written consent was obtained from each household’s head to conduct the survey. All individuals residing in the study area were screened for mycetoma by careful clinical examination after verbal consent.

### Diagnosis of mycetoma patients

All individuals with swelling involving any part of the body or sinus formation with or without grains were classified as suspected mycetoma cases. All suspected cases were referred to Wad Onsa Mycetoma Satellite Centre, where they were clinically examined, and an expert radiologist performed lesional ultrasound (US) examinations to ascertain a mycetoma diagnosis. Confirmed mycetoma patients were defined as individuals with swelling in any part of the body with or without sinus formation, multiple sinuses with or without grain discharge that was evident by ultrasound examination in the form of a pocket of fluid containing echogenic grains [17].

Data collection was done using electronic questionnaires, and it included suspected cases’ demographic data (age, gender, marital status, educational level, and occupation) to describe the characteristics of the targeted population. The clinical data related to mycetoma collected were onset of the disease, lesion site, history of trauma and family history. For the behavioral practices, data on shoe-wearing and direct contact with animals and thorny trees were collected in form of practicing arable farming which deals with cultivating crops and animal grazing. Animal grazing is considered a farming activity where domestic livestock are allowed outdoors to roam around and consume wild vegetations. The data were collected by trained medical doctors who received training on the data collection process and consent form administration.

### Data analysis

Data were sent directly to a server at the Mycetoma Research Centre, Data Centre, Khartoum and visualised using Microsoft Excel (Microsoft Corp., Redmond, WA). Data verification, cleaning and analysis were done using Statistical Package for Social Sciences, SPSS 25 (SPSS, Chicago, IL, USA). Descriptive statistical analysis was performed to calculate the overall prevalence and to determine the individual data variables. Maps for mycetoma prevalence were developed using ArcGIS 10.5 ([ESRI] Inc., Redlands CA, USA).

## Results

**Fig 2:**
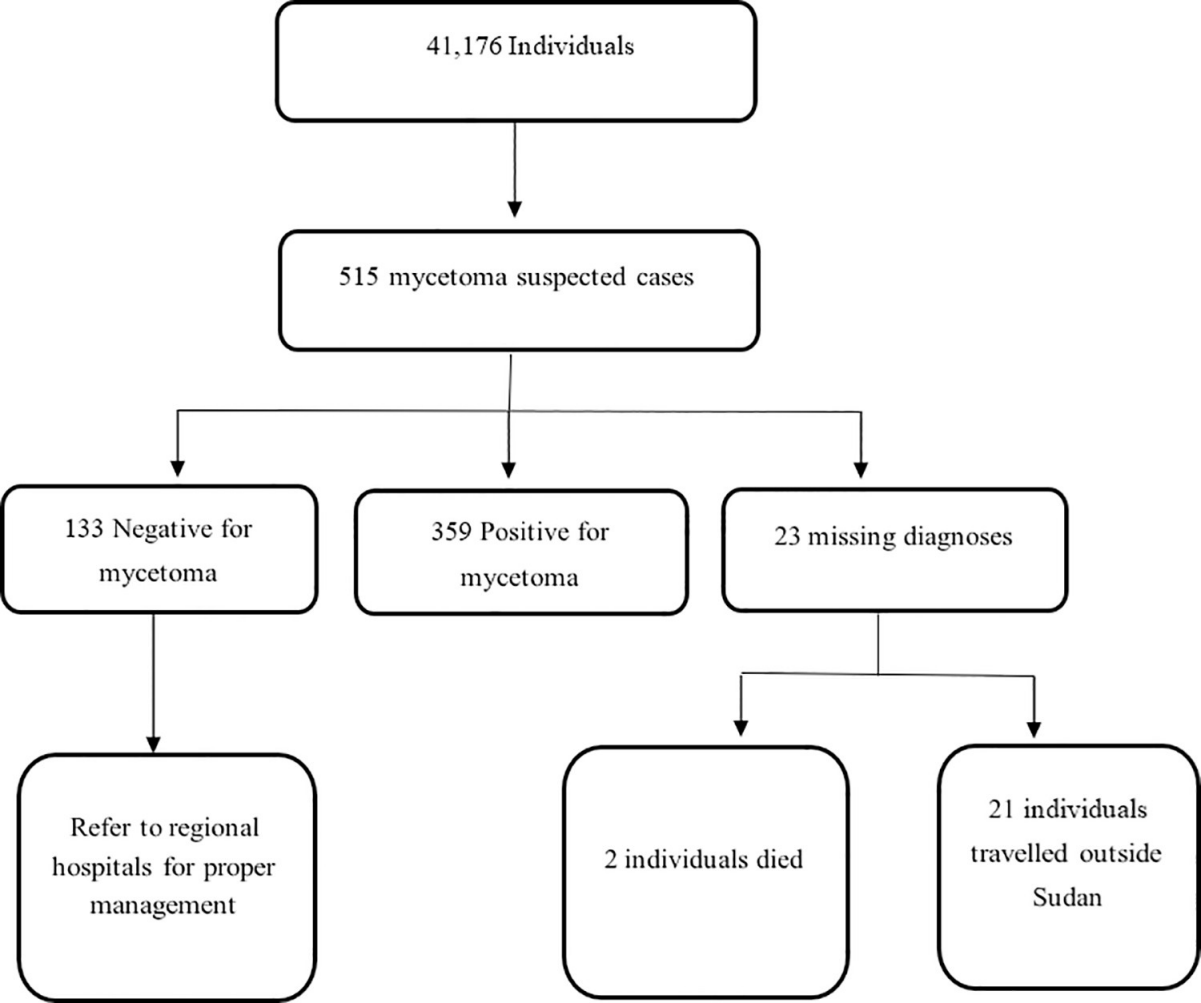
Flow diagram of the total population covered in the survey and the suspected cases identified after clinical examination. It also describes the category of the patients after ultrasound examination.

A total of 41,176 individuals were surveyed, from which 515 suspected mycetoma cases were detected. Of these, 359 patients (69.7%) proved to have mycetoma, the diagnosis was not confirmed in 133 (25.8%), and 23 cases did not attend for confirmation of the diagnosis. (Fig 2)

The median age of the participants was 17 years (interquartile range [IQR], 8 to 34). The maleto female ratio of the respondents in the population of the study was 1.0:1.0. The sex ratio of the cases was male to female; 0.9:1.0. Only 36.2% experienced pain, and 35.7% had a family history of mycetoma. Students in the age group of 10–20 years were the commonest and constituted 19.2% of mycetoma cases, followed by farmers and shepherds (17.5%). (Table 1)

**Table 1:** The demographic features of mycetoma cases seen in Eastern Sennar Locality, Sennar State, Sudan. (N = 359 mycetoma cases and 41,176 surveyed individuals).

Variable	No. (%)
**Visible swelling**	
Yes	290 (80.8%)
No	69 (19.2%)
**Visible sinuses**	
Yes	138 (38.4%)
No	221(61.6%)
**Discharge**	
Grains	115 (32%)
Fluid, pus, blood	7 (1.9%)
No discharge	237 (66%)
**Grains**	
Black	112 (97.4%)
White	3 (2.6%)
**Site of the lesion**	
Upper extremities	94 (26.2%)
Lower extremities	245 (68.2%)
Head, neck, trunk, back and perineal area	14 (3.9%)
Multiple sites	5 (1.7%)
**Pain**	
Yes	130 (36.2)
No	229 (63.8%)
**Family History**	
Yes	128 (35.7%)
No	231 (64.3%)

**Fig 3:**
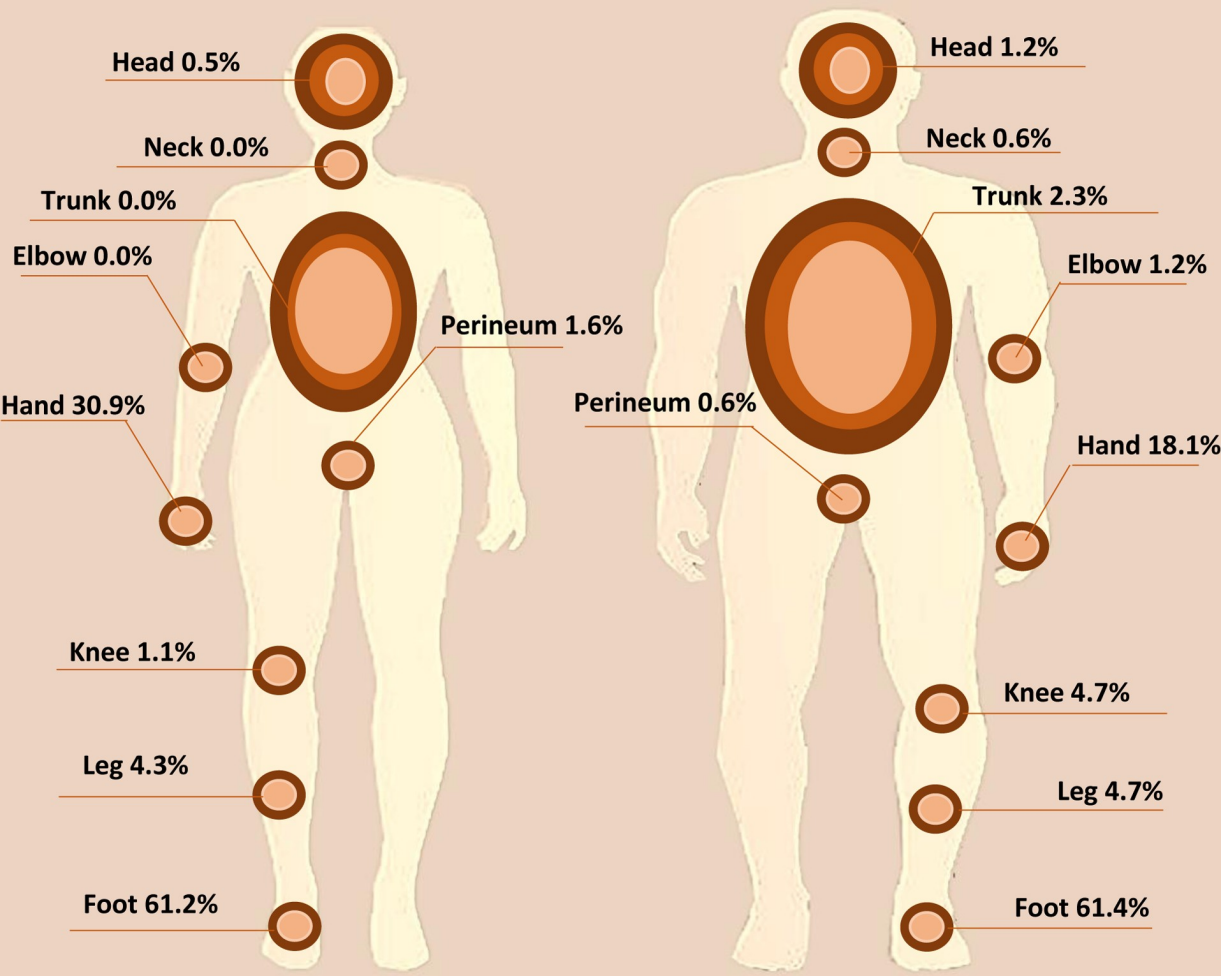
Physical distribution of mycetoma lesions for female (left side) and male (right side) cases. The feet constituted more than 60% of the sites of the lesion in both males and females. The fewest lesions were recorded on the neck and perineum for males and on the neck, trunk, and elbows.

Most of the lesions were on the lower extremities (68.2%), followed by the upper extremities (26.2%), and 1.7% of patients had multiple lesions at different sites. Females had more lesions in the upper extremities than males with a percentage of (58.5%). (Fig 3)

Most of the patients (80.8%) had swelling, 38.4% had sinuses, 33.9% had discharging sinuses only following previous surgical excisions, 32% had grains discharge, and 97.4% of the grains were black.

The study showed that most houses, 334 (93%), had soil or sand floors, and most of them (83%) had roofs made of traditional material such as tree branches and palm leaves. Surrounding walls, when present, were made of mud and animal dung (29.8%), red bricks and concrete (27.7%) or tree branches (8.6%), and 33.7% had no surrounding walls.

More than half (51%) of the mycetoma patients owned animals, and 34.8% raised animals within the household. The study showed most of the cases (64.9%) practised arable farming while only 29.5% practised animal grazing (rearing).

**Fig 4:**
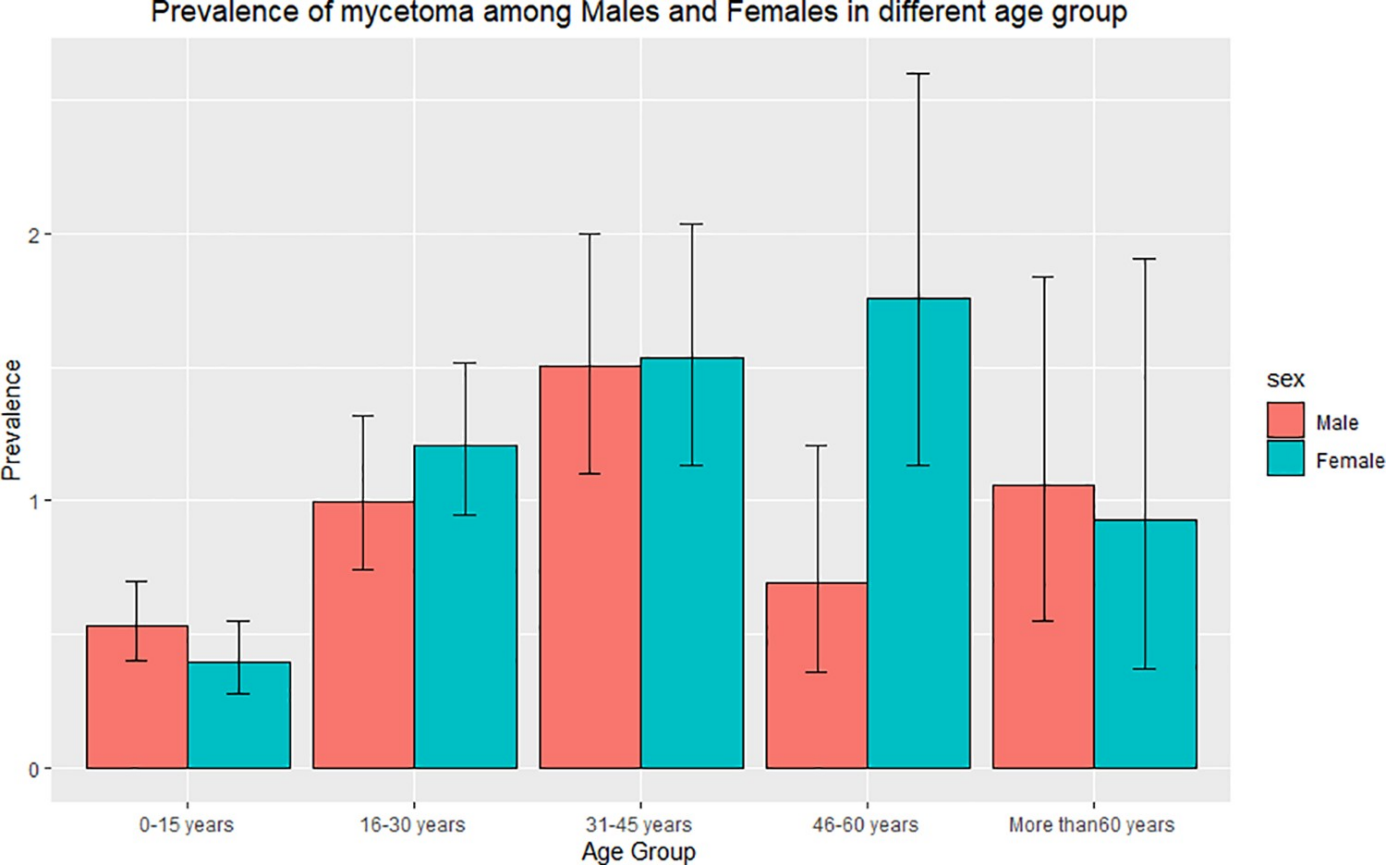
Bar plot of the prevalence of mycetoma among different age groups according to gender. Males had a higher prevalence among the age groups 0–15 years and more than 60 years, and females recorded a higher prevalence among the other age groups.

The overall mycetoma prevalence was 0.87% (95% CI = 0.78–0.97%), the prevalence among males was 0.83% (95%CI = 0.71–0.96%), and among females was 0.92% (95% CI = 0.79–1.06%). The mycetoma prevalence was highest in the age group 31–45 years (1.52%, 95% CI = 1.23–1.86%) followed by the age group 16–30 years (1.11%, 95% CI = 0.93–1.33%). Most of the males with mycetoma were in the younger age groups compared to females. (Fig 4)

Married and illiterate individuals had a higher mycetoma prevalence (1.34%, 95% CI = 1.16–1.53%) and (1.24%, 95% CI = 1.04–1.48%) respectively. Prevalence was higher among individuals with a history of trauma (1.63%, 95% CI = 1.36–1.95%). Wearing shoes did not affect the mycetoma prevalence as individuals who wore shoes most of the time had a prevalence of 0.94% (95% CI = 0.84–1.06), while individuals who wore shoes either at home or work only had a prevalence of 0.74% (95% CI = 0.50–1.08). (Table 2)

**Table 2:** Prevalence mycetoma patients (N = 359) among the studied individuals (41,176) in Eastern Sennar locality, Sennar State, Sudan.

Variable	Cases	Population	Prevalence %(95%CI*)
**Gender**			
Male	172	20753	0.83 (0.71–0.96)
Female	187	20423	0.92 (0.79–1.06)
**Age group**			
0–15 years	90	19227	0.47 (0.38–0.57)
16–30 years	121	10859	1.11 (0.93–1.33)
31–45 years	93	6111	1.52 (1.23–1.86)
46–60 years	36	3094	1.16 (0.82–1.61)
>60 years	19	1885	1.01 (0.61–1.57)
**Marital Status**			
Currently married	198	14830	1.34 (1.16–1.53)
Currently unmarried	161	26346	0.61 (0.52–0.71)
**Education**			
Literate	208	23805	0.87 (0.76–1.00)
Illiterate	124	9976	1.24 (1.04–1.48)
Underage of school	27	7395	0.37 (0.25–0.53)
**History of trauma**			
Yes	123	7527	1.63 (1.36–1.95)
No	236	33649	0.70 (0.61–0.80)
**Wearing shoes/ slippers**			
Both work and home	264	28027	0.94 (0.84–1.06)
At work or home only	27	3647	0.74 (0.50–1.08)
Not at all	68	9502	0.72 (0.56–0.91)

*CI = Confidence interval

### Geographical distribution of mycetoma

The study included sixty villages distributed among all the administrative units of the locality. Doba had the highest prevalence of mycetoma among all the administrative units (1.14%), and Elreif Elshargi had the lowest (0.15%). The highest village prevalence was recorded in Awlad El-Tai village (6.2%), followed by Wad Yagoub (4.9%), and the lowest prevalence was estimated in Kasab Garbi (0.11%). There were nine villages with no cases and an average of seven cases per village across the other 51 surveyed villages (range 1–39). The mycetoma prevalence map showed cases clustered within the central and north-eastern part of the locality, while the south-western part had few or no cases. (Fig 1) In the figure, more than nine villages have a prevalence of 0.00, and that was because eight villages recorded only one case.

## Discussion

Mycetoma is one of the neglected tropical diseases that is increasingly recognised by the international scientific and funding communities. Most of its epidemiological characteristics are an enigma [18]. Globally, its incidence and prevalence are not well known. Furthermore, the infection route, incubation period and factors contributing to susceptibility and resistance to mycetoma are not well documented [19]. This is due to a lack of international attention, research funding, and interested institutes to work on mycetoma, leading to a scarcity of data on the disease’s basic epidemiological features and its seriousness and magnitude, promoting the negligence cycle. Furthermore, due to the patients’ low socio-economic and health education levels, the painless and slow-progressing nature of the disease, the lack of health facilities in endemic regions and the patients’ inability to reach central hospitals for management, they tend to present late with advanced disease [3]. Hence, most of the mycetoma epidemiological characteristics were obtained from case reports and a series of hospital patients with advanced disease, representing the tip of the iceberg. The present study is distinctive as it is community-based, and multi-level data were collected to determine the mycetoma clinical epidemiological characteristics in the study area.

This study documented a mycetoma prevalence of 0.87% in the studied locality, higher than previous Sudan estimates [20]. Abbott studied individuals who managed to reach health facilities for diagnosis and reported a disease prevalence of 0.51% among hospital patients seen in Khartoum during a study period of 36 months. In addition to higher prevalence in Atbara, Ed Dueim, and Wad Madani cities (within central Sudan states) with estimates of 0.92%, 0.93% and 1.18%, respectively and still the reported prevalence could have underestimated the actual burden of disease [14]. The reported prevalence (1.45%) by Fahal and associates in 2014 is higher than that reported in the present study. That could be attributed to the high endemicity of mycetoma in the village where that survey was conducted [15].

Van de Sande in 2014 conducted a systemic review in an attempt to determine the global burden of mycetoma, reviewing 8,763 cases from different countries around the world, and estimated the prevalence for endemic countries such as Sudan and Mexico to lie between 0.0015 and 0.018 cases per 1000 inhabitants [20].

Previous studies suggested that the distribution of mycetoma is affected by environmental and climate factors [21]. A modelling study predicted mycetoma occurrence in central and south-eastern states of Sudan and along the Nile river and indicated that arid areas proximal to water sources, soil with low concentrations of calcium and sodium and areas with a variety of thorny tree species provided the most suitable environment for the occurrence of mycetoma in Sudan [22].

Our results showed that mycetoma is prevalent in the central and north-eastern locality, with Doba administrative unit having the highest prevalence recorded. The local environmental and sanitation conditions and may explain this geographical distribution [8,17]. People in those areas mainly work in farming and with animals, are exposed to organisms residing in soil and lack access to good sanitation. These factors are all likely to promote susceptibility to mycetoma.

Male predominance is a documented feature of mycetoma. In most studies, the reported male/female ratio is 3–4: 1 [23,24]. In this study, the disease prevalence was slightly higher in females, contradicting all the previous reports. The prevalence reported here may be more accurate as it is a community-based study, and most of the previous studies were hospital-based. Females are less likely to seek medical treatment and often present late, suggesting previous studies may have been biased by female health-seeking behaviour [4,19].

In this study, individuals in the age group 16–45 years were most affected. This concurs with the literature, and the results are not surprising given that this is the most active group in society and is often involved in farming and animal grazing practices [25–27]. A fifth of the affected individuals were students who help with farming and animal care during vacations. Also, students have to walk long distances to and from schools in the rural communities and hence are more exposed to the environment.

The medical literature documented a high incidence of mycetoma among farmers and labourers. It was postulated that the direct and continuous contact with the environment and soil where the causative organisms reside and minor trauma and thorn pricks are important disease predisposing factors [9]. In the present study, arable farmers had a higher prevalence of mycetoma compared to shepherds. Furthermore, individuals with a history of local trauma and thorn pricks had a higher mycetoma prevalence, which is in line with the reported studies [21,28,29].

Several studies suggested a possible role of animal dung in causing mycetoma as some organisms were isolated from the dung, which may act as a reservoir for them [21,30–32], allowing the direct transmission to the human. Most of the individuals living in rural areas are in direct contact with animals such as cattle, donkeys, dogs, sheep and chickens. We found no strong evidence supporting a role for animals or their dungs in the development of mycetoma, which needs further in-depth study [19,31].

In this study, the disease duration ranged between one month and 40 years, with a mean duration of 4.5 years before presentation. A study conducted in West Bengal showed that the disease period could vary according to causative organisms such as Nocardia, Streptomyces, Actinomadura species, *and Madurella*. *grisea*. The least duration was three months before presentation for all organisms and three years *for Nocardia* and *Actinomadura species*, while *Madurella grisea* organisms reached up to nine years [24]. However, due to the painless nature of the disease, duration is subject to memory bias.

Mycetoma was reported to affect different body parts, but the foot and hand are affected the most [33]. In this study, the obtained data align with this: the lower extremities (67%) and upper extremities (22.6%) were most affected. This result is expected since men mostly work barefooted farming, allowing exposure to injury and inoculation with mycetoma-causative organisms. In this study, only 2.3% of male cases presented with trunk mycetoma, which could be attributed to the nature of rural residents’ occupation that makes them prone to injuries in upper or lower extremities. In contrast, trunk mycetoma was recorded in 19% in a study conducted in Mexico for mycetoma patients recorded in mycological centres from 1958 to 2012 and 10% from a single centre study in patients recorded in the period between 1980 to 2013 as they consistently carry woods for domestic activities on their backs [34,35].

An interesting observation was noted in this study, that females have more hand mycetoma. This can be attributed to the fact that females are commonly responsible for cooking and getting wood from forests to be used as a fuel source and are therefore prone to minor hand injuries. Also, they are involved in different indoor activities such as cleaning the floor and removing animal dung from within the houses where the organisms possibly reside.

The triad of subcutaneous swelling, multiple sinuses and discharge that contain grains is pathognomonic of mycetoma [1,36,37]. In our study, 80.8% of the patients presented with swelling, 38.4% had sinuses, and 32% of these sinuses produced purulent and sero-purulent discharge with mostly black grains. Black grains are usually produced in the fungal form of the disease eumycetoma, and in Sudan, eumycetoma accounts for 60–70% of mycetoma cases and the same pattern is observed for patients in Eastern Sennar locality [15]. Only 3% had white grain discharge, which could be attributed to actinomycetoma, the bacterial type of mycetoma. Actinomycetoma is not widespread in Sudan but constitutes the majority of mycetoma cases in Central and South America [20,34].

Currently, there is no evidence-based preventive or control programme or notification system for mycetoma as most of its epidemiological characteristics are not well known. Wearing shoes is considered a preventative measure for mycetoma since mycetoma is most commonly seen in the foot and is believed to be contracted via injuries by sharp objects [28].

In this study, we found that wearing shoes did not affect the prevalence of mycetoma in the region, as people who wore shoes had almost the same prevalence as people that did not wear them. However, in mycetoma endemic regions, people frequently work in the fields and walk barefooted as the available shoes can be an obstacle to executing these activities and sometimes are regarded as a hindrance. Due to the hot weather in Sudan, people tend to wear light open shoes, which offer little foot protection.

Even though individuals living in endemic areas share the same environment and are exposed to similar risk factors, only a proportion develop mycetoma. This supports the hypothesis that genetic factors play a role in mycetoma. In our study, 35.7% of mycetoma patients had a family history of mycetoma. It is important to highlight that consanguineous marriage are common in rural areas where mycetoma is endemic, which may explain this observation. In previous studies, family history was strongly associated with disease recurrence; individuals with a positive family history might be more genetically susceptible to contracting the disease initially and getting recurrent disease [38].

In conclusion, this clinical epidemiological community-based study is the first of its kind to be reported. The disease prevalence reported here may be more accurate as it was generated from a large study population. It revealed an equal sex ratio which contradicts most of the previous reports. Other findings are in line with those reported previously. Further epidemiological studies are needed to determine mycetoma prevalence in Sudan and bridge the gaps in our understanding of the epidemiology of mycetoma, which is vital to design evidence-based control and prevention programmes. Furthermore, these surveys are also helpful in early case detection and treatment, health education, disease awareness, and advocacy, reducing the disease burden and improving the disease prognosis. However, implementing surveys in rural areas in Sudan could be difficult, particularly during the rainy seasons, and proper team training, good facilities and collaboration between different stakeholders are all required.
